# Hybrid Technique of Lamellar Keratoplasty (DMEK-S)

**DOI:** 10.1155/2013/254383

**Published:** 2013-06-05

**Authors:** Pavel Studeny, Deli Sivekova, Katerina Liehneova, Magdalena Vokrojova, Pavel Kuchynka

**Affiliations:** Ophthalmology Department, Medical Faculty of Charles University and Teaching Hospital Kralovske Vinohrady, Srobarova 50, Prague 100 34, Czech Republic

## Abstract

*Purpose*: To evaluate the outcomes of the hybrid technique of posterior lamellar keratoplasty (DMEK-S). *Materials and Methods*: 71 eyes of 55 patients enrolled in a single-center study underwent posterior lamellar keratoplasty with a hybrid lamella DMEK-S implanted using a solution implantation technique, owing to endothelial dysfunction. The outcome measures studied were visual acuity and endothelial cell density. *Results*: The rate of endothelial cell loss caused by surgery was 43.8%. During followups, we observed the stabilization of postoperative findings, or at minimum a very low rate of corneal endothelial cell loss. The UCDVA and BCDVA dramatically improved postoperatively. The rebubbling rate in our group of patients was 61.9%. We replaced the lamella due to its failure or malfunction in 17 patients (23.9%). *Conclusion*: In summary, DMEK-S combines the advantages of DSEK/DSAEK and DMEK. The central zone of bare Descemet's membrane and endothelium allows for very good visual outcomes, and the peripheral rim allows for better manipulation of the lamella during implantation. It is an effective method of treating the endothelial dysfunction of various etiologies, but the high complication rate needs to be addressed before widespread implementation of the technique in the future.

## 1. Introduction

Corneal endothelium dysfunction, as well as the resulting reduced transparency due to corneal edema, remains a major indication for corneal transplantation. Until 1998, the only known technique for exchanging the corneal endothelium was a full-thickness corneal transplantation—penetrating keratoplasty, even though this type of disease affects only a thin inner layer of cells—the endothelium. In 1998, Dr. Melles published results from the first successful transplantation of the posterior corneal layer—posterior lamellar keratoplasty (PLK) [1]. Its main advantages compared to conventional penetrating keratoplasty are a rapid improvement in visual functions, lower incidence of serious postoperative complications, a sutureless technique and significantly higher comfort for the patient [1, 2]. The disadvantages of these surgeries are the relatively high technical difficulty involved and the high loss of transplanted endothelium cells during the procedure in the early postoperative period [3, 4]. Therefore, many eye surgeons have focused on this issue in an attempt to simplify the procedure and improve the long-term results of lamellar keratoplasty. The optimal procedure has not yet been clearly established and there are many variations of the operation.

### 1.1. Endothelium-DM-Posterior Stroma Lamella

The first type of lamella is composed of endothelium, Descemet membrane (DM), and a part of the deeper stroma, which works as a structural support to enable feasible manipulation of the lamella and to prevent massive damage of the fragile endothelium. Such techniques are called Descemet's Stripping Endothelial Keratoplasty (DSEK), if the lamella is created manually, or Descemet's Stripping Automated Endothelial Keratoplasty (DSAEK), if the lamella is made using a microkeratome or femtosecond laser [5–7]. The main advantages of these techniques are relatively easy manipulation, less stress on the endothelial cells, and good recognition of the stromal and endothelial side of the lamella into the anterior chamber of the recipient's eye. A lamella with stroma keeps a convex shape, and it is also possible to mark the stromal side. The main disadvantages are the plus of the stromal tissue and possible interlamellar problems, so the best corrected visual acuity is very often slightly decreased [3, 6, 8–10].

### 1.2. Endothelium-DM Lamella

The second type of lamella consists of DM with endothelium, while the stromal support is absent. The donor discs are prepared from the corneoscleral donor button by stripping a circular portion of tissue; the transplantation technique has been referred to as Descemet Membrane Endothelial Keratoplasty (DMEK) [11, 12]. The preparation of the recipient corneal bed is the same as in a DSEK lamella. A donor button (9.5 mm) is trephined from the endothelial side and subsequently stripped from the posterior stroma. In the storage medium, the lamella spontaneously forms a roll with the endothelium on the outside. The endothelium-DM roll may be evaluated after treatment with trypan blue and sucrose. The preparation technique is standardized, and its basic parameters, as well as the postoperative follow-up, have been repeatedly published. These techniques are hypothetically optimal, because the surgeon replaces the involved endothelium and DM with the same portion of tissue. In this approach, there are no problems with interlamellar opacities, and the vision after surgery is very often excellent; unfortunately, however, manipulating such a thin lamella is difficult; in particular, the unwrapping of the lamella at the insertion may be quite stressful on the endothelium. 

### 1.3. Endothelium-DM-Stromal Support Lamella

**Figure 1:**
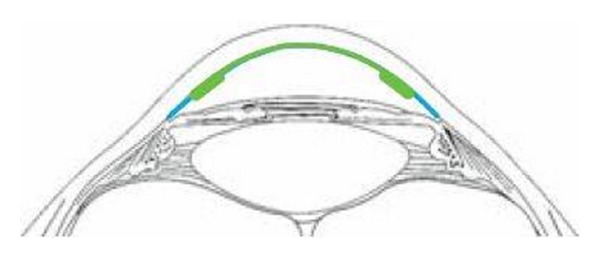
DMEK-S scheme.

The third type of lamella has a thicker periphery, similar to the lamella in DSEK or DSAEK, and a central part that consists of bare DM and endothelium, as in the DMEK lamella. The techniques by which this lamella is prepared can be called *hybrid techniques*, because they combine the advantages of both the previous techniques. The central part without stroma insures excellent optical results after surgery, comparable with those of successful DMEK patients (Figure 1). The stromal rim fixes the thin, fragile central part, helping to maintain its shape and preventing the scrolling of the DM. Moreover, the stromal part allows the anterior-posterior orientation of the lamella to be marked. This enables the surgeon to know exactly on which side the endothelium is located when manipulating the lamella, as well as its orientation in the anterior chamber. We called this special type of surgery DMEK-S (Descemet Membrane Endothelial Keratoplasty with Peripheral Stromal Support) [13].

This paper describes the surgical procedure, implantation technique, and visual outcomes in each group of patients after DMEK-S surgery.

## 2. Material and Methods

Seventy-one eyes of 55 patients enrolled in a single-center study underwent posterior lamellar keratoplasty with a hybrid lamella DMEK-S implantated using a solution implantation technique, owing to endothelial dysfunction. The study was held in the Ophthalmology Department of Kralovske Vinohrady Teaching Hospital and the 3rd Medical Faculty of Charles University, Prague, from 2009 through 2011. There were 10 men and 45 women. The mean age of the patients was 71.25 ± 12.6 years (range 40–94 years). Patients who did not have adequate follow-up results were excluded. The predominant indications for posterior lamellar keratoplasty were pseudophakic or aphakic bullous keratopathy (27 eyes), Fuchs' endothelial dystrophy (15 eyes), and posterior polymorphous corneal dystrophy (7 eyes). In 22 eyes, the surgery was performed due to previous graft endothelial failure (Table 1). Sixteen eyes underwent DMEK-S combined with cataract extraction and PC IOL implantation. All surgeries were performed by a single surgeon.

### 2.1. Preoperative Care

Preoperative medication routinely consisted of antibiotic-steroid combination drops applied 5 times daily, 2 days preoperatively. Patients applied Tobradex (Alcon), which contains tobramycin and dexamethasone. If the patient was using antiglaucomatous medication, he continued using it until the day of surgery. The surgery was performed under an inpatient department regimen; the duration of hospitalization was about 4 to 7 days. The proper preoperative preparation on the day of surgery was the same as in other anterior segment surgeries. To induce preoperative pupil dilation, 0.5% tropicamide drops were used. The surgeries were performed with topical anesthesia using local anesthetic drops (bupivacaine, lidocaine). In the case of single-procedure DMEK-S and cataract surgery, topical anesthesia was combined perioperatively with intracameral anesthetics (lidocaine) according to need. In the operating theatre, the standard procedure for disinfection was followed, as was periocular and eyelid skin disinfection, and conjunctival sac and corneal surface irrigation, using a 5% Povidone Iodine solution (Betadine, Egis Pharmaceuticals) for 3 minutes. 

### 2.2. Surgical Technique

#### 2.2.1. Donor Lamella Preparation

We obtained the tissue used for donor lamella preparation before the DMEK-S surgery from the International Eye Tissue Bank OTB01 in Prague, Czech Republic. The 17 mm corneoscleral buttons were held in an EUSOL medium with an expiry date 14 days after the death of the donor. The only criterion when selecting a suitable donor was high-quality endothelium. We consider 2500 cells/mm^2^ to be the minimum required endothelial cell density prior to the preparation of lamellae. All lamellae were prepared in the operating room under sterile conditions just prior to implantation. 

We placed the corneoscleral disc endothelium side up in the Barron artificial chamber (Katena) and separated the Descemet membrane from the stroma using the “big bubble” technique. We injected an insulin needle into the corneal stroma in the outer periphery of the donor disc to minimize damage to the transplanted endothelium. Air was subsequently injected into the stroma that caused the whitish appearance of the cornea, after which a central air bubble was formed, which separated the Descemet membrane from the stroma. Given that the corneal endothelial side was at this stage placed upwards, the forming bubble could be monitored visually. We covered the endothelial surface with a fine layer of dispersive viscomaterial (Viscoat, Alcon), turned the donor cornea, and placed it securely in the artificial chamber, endothelium downwards. We created adequate pressure in the artificial chamber using the connected service syringes.

We completely removed the stroma from the central 6 mm zone dissecting the big bubble and left the deep stromal portion in the periphery of the donor cornea using a crescent knife. We used a color marker (Skin Marker, Kendall) to mark the lamella in the peripheral area with retained deep stromal layers, enabling the next phase of the operation, which was to identify the stromal and endothelial sides of the disc. For this purpose, we chose either laterally asymmetric letters (S, F) or two different consecutive symbols (one point, two points). After this, we used an 8.5 mm Barron punch for trephination of the lamella. The preparation of the lamella usually took about 10–15 minutes (See the Video in Supplementary Material available online at http://dx.doi.org/10.1155/2013/254383).

In four cases, we observed rupturing in the Descemet membrane caused by rapid big bubble formation during preparation. In these cases, we used a standby donor cornea. In eight cases, there was no detachment of the Descemet membrane and no big bubbles were created. In these cases, we proceeded with gradual manual preparation of the stromal lamellae. In five corneas, we reached the Descemet membrane without rupturing it, but in three cases, a microperforation of the Descemet membrane occurred; therefore no other preparation was performed, and the lamella was left with deep stromal layers and implanted into the eye in this condition. These three patients were excluded from the evaluated group of patients. 

#### 2.2.2. Surgical Preparation of the Recipient's Eye for the Transplantation of the Lamella

We performed a 2.75 mm corneal tunnel incision at 12 O'clock in the limbus using a single-use knife; We then made 2 side ports at 3 and 9 O'clock, respectively, in the limbus using a paracentesis knife. If there was any significant corneal edema preventing sufficient visualization of the anterior chamber structures, we performed epithelial abrasion. This procedure was chosen for 14 patients. In these cases, we covered the cornea with a bandage contact lens at the end of the surgery, and it was left in the eye until the epithelium healed. 

We used the technique of continual descemetorhexis to remove the patient's Descemet membrane and damaged endothelial cells in a central area of approximately 9 mm. To maintain the anterior chamber during this procedure, we used an irrigation cannula from the phacoemulsification machine (Katena), introduced through the left paracentesis and connected to the infusion of a Ringer solution (Fresenius Kabi). We implanted the donor lamella in the eye so prepared. 

In patients where corneal transplantation was combined with cataract surgery, we filled the anterior chamber with a cohesive viscoelastic material (Provisc, Alcon) after making an incision. Using capsular forceps, we performed continuous circular capsulorhexis. Subsequently, we performed the descemetorhexis and followed to completion the standard phacoemulsification cataract surgery technique and posterior chamber IOL implantation. The anterior chamber was properly flushed after the complete of the cataract surgery using irrigation and aspiration cannulas to completely remove the residual viscoelastic material. The subsequent process was identical to that of a single procedure surgery.

#### 2.2.3. Implantation of the Lamella

We implanted the lamella using our own technique utilising Balanced Salt Solution (BSS) flow and a plastic cartridge, which we named Solution Implantation Technique (SIT). We introduced this implantation technique into our practice in December 2008. Our aim was to speed up and simplify this part of the surgery and to minimize the burden on endothelial cells during implantation.

The endothelial side of the lamella is covered with a small amount of dispersive viscoelastic solution (Viscoat, Alcon) and folded approximately 50 : 50, endothelial side inwards. To transport the tissue into the open entrance of the cartridge, we held the peripheral stromal part of the lamella with forceps. We pulled the tissue into the closed part of the cartridge using a small amount of pressure with a cyclospatula on the stromal rim. We then closed and connected the cartridge to the end of an infusion tube set with a syringe filled with BSS. We used an irrigation cannula to maintain the anterior chamber during the implantation. The irrigation pressure has to be low so as not to push the lamella out of the eye. The surgeon held the irrigation cannula in his nondominant hand (left in our case) and inserted it through the paracentesis; the surgeon held the cartridge containing the rolled lamella and connected with the syringe in his dominant hand. The opening of the cartridge was inserted into the main incision (3.0 mm.). At this moment it is very important to switch off the irrigation by releasing the pedal, so as not to overpress the lamella deeper into the cartridge. With a small amount of pressure on the syringe, we pushed the BSS carrying the lamella into the anterior chamber. The lamella spontaneously unrolled due to the peripheral rim. We removed the implantation device and the irrigation cannula, and with repeated amounts of small pressure on the cornea, using a cannula for instance, we could centrate the lamella. After this centration, the lamella was fixated to the recipient cornea using an air bubble. We left the anterior chamber completely filled with the air bubble for 60 minutes, with the patient lying in the supine position in the operating theatre. Afterwards, we replaced part of the air using BSS, so the remaining bubble was still covering the edges of the lamella (See Supplementary Video).

We checked the water tightness of the corneal wounds and performed hydration of the supplement paracentesis. At the end of the operation, we applied combined antibiotic and corticosteroid eye drops (Tobradex, Alcon). We covered the eye with a transparent plastic cover; the patient was transferred to the inpatient department and advised to maintain the correct, supine position, that is, looking up at the ceiling. The air bubble in the anterior chamber was left to its spontaneous absorption, that is, about 2-3 days.

### 2.3. Postoperative and Follow-Up Care

In the postoperative period, we biomicroscopically observed the position and attachment of the lamella. If the lamella was detached after complete air bubble absorption from the anterior chamber, we performed rebubbling. This was usually done 7 days after the principal surgery in the operating theatre under sterile conditions and an outpatient regimen. The anterior chamber was completely filled with the air bubble and the patient was kept for 60 minutes in the same position on his back. After one hour, the air was partially replaced with Ringer solution so that the edge of the air bubble covered the borders of the lamella. If, despite this procedure, the lamella was still detached, rebubbling was repeated again after one week. If the lamella was not attached even after repeated air-bubble-filling (after a maximum of 3 times), the surgery was assessed to be unsuccessful and the patient was indicated for retransplantation, but only after a period of at least three months after principal surgery. 

In the postoperative period, patients applied topical antibiotic (tobramycin) and steroid (dexamethasone) combined drops (Tobradex, Alcon) 5 times daily for two weeks after the surgery. Two weeks postoperativly, the patients started to apply only the local steroid drops (Dexamethasone, WZF Polfa) 3 times daily and continued this for a 6-month period. After six months the steroid drops were replaced with nonsteroid antiphlogistic indomethacin (Indocollyre, Laboratoire Chauvin S.A.), which was applied 3 times daily. When faced with an increase in intraocular pressure, local medication was combined with antiglaucomatous drops (timolol, dorsolamide).

### 2.4. Evaluation

**Figure 2:**
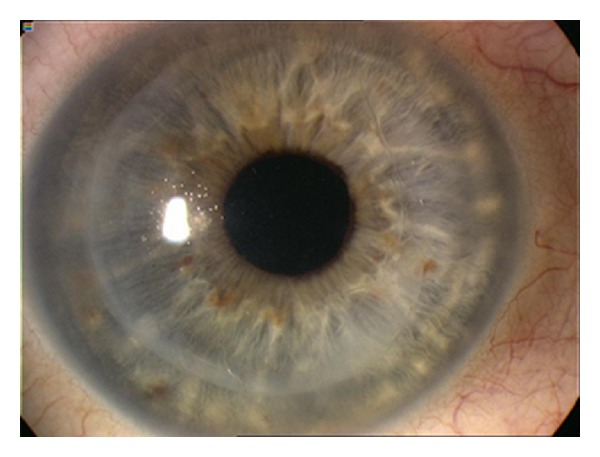
Properly attached DMEK-S lamella.

We recorded the number of peroperative and postoperative complications. In each patient, we noted the number of further air injections into the anterior chamber needed to properly attach the donor lamella (Figure 2).

In the postoperative period, we monitored the value of intraocular pressure using noncontact tonometry, corrected and uncorrected visual acuity of the Snellen optotypes 1 month, 3, 6, 12, 18, and 24 months after surgery, and corneal endothelial density using a noncontact specular endothelial microscope (Topcon SP 3000) at 6, 12, and 24 months.

Since the endothelial cell replacement is the main objective of this type of corneal transplantation, the endothelial cell density (ECD) data best indicates the surgical success rate of this surgery. We evaluated endothelial cell loss caused by the surgery itself by comparing the ECD data, declared by the eye tissue bank in the document accompanying the transplanted tissue, with postoperative ECD in patients with transplanted endothelium. Subsequently, we evaluated any further loss of endothelial cells over time. 

The corrected and uncorrected visual acuity indicated the clinical success rate of this surgery and, primarily, the benefit that it brings to the patient. Visual acuity was examined using projection optotypes and recorded using decimal numbers.

We evaluated the statistical significance of the results using both a Student's paired *t*-test and a multidimensional test (Bonferroni test).

## 3. Results

The study involved 71 eyes demonstrating endothelial dysfunction of various etiologies, but without other ocular pathologies that could affect proper surgery or postoperative results of visual acuity.

**Figure 3:**
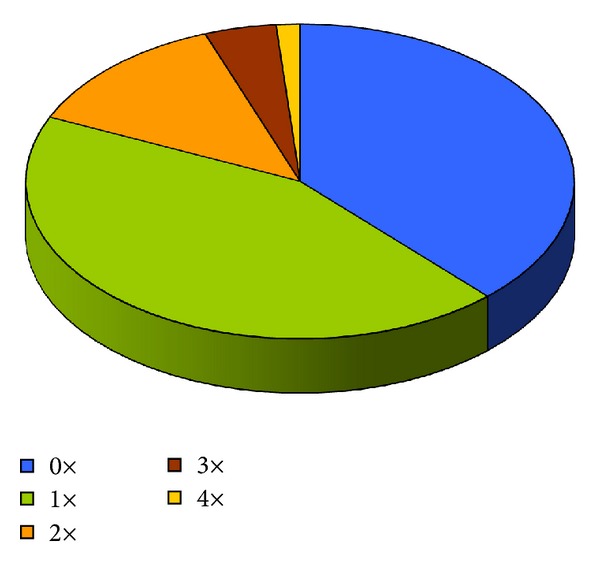
Postoperative rebubbling rate.

In our group of patients, we realized primary lamella attachment in 27 eyes; in 31 eyes we, performed one rebubbling; in 9 eyes we repeated the air-bubble filling twice—in 3 cases 3 times and in 1 case 4 times (Figure 3). In cases involving total detachment of the lamella we, performed only 3 rebubblings according to our internal criteria. Empirically, we concluded that in these cases further rebubbling would have been useless.

We had to replace the lamella due to its failure or malfunction in 17 patients (23.9%). We picked five explanted lamellae at random and evaluated them histologically. There were only a few endothelial cells left on the explanted lamella. Therefore, we supposed that endothelial loss was one of the causes of unsuccessful surgery. We decided to exchange the lamella in 10 patients within 3 months following primary surgery, in 5 patients within 6 months, and in the remaining 2 patients within a year. In these cases, repeated DMEK-S surgery was indicated [7, 14]. These patients were no longer included in our study. In all 17 cases, the repeated surgery was successful, with comparable visual and endothelial outcomes as those presented in the main group of patients.

### 3.1. Endothelial Cell Density (ECD)

The average preoperative endothelial cell density of the donor graft was 2907 ± 51.9 cells/mm^2^. Donor characteristic data were declared by the eye bank. We analyzed the ECD in a 1-month postoperative follow-up, and the average endothelium cell count was 1273 ± 82.7 cells/mm^2^, which meant an endothelial cell loss of 43.8%. During further follow-ups in months 3, 6, 12, 18, and 24, we observed a stabilization of postoperative findings or at most a very low rate of corneal endothelial cell loss. The mean ECD was 1177 ± 78.6 in the 3-month follow-up, 1108 ± 72 in the 6-month follow-up, 1005 ± 79.9 in the one-year follow-up, 1163 ± 145.9 in the 18-month follow-up, and 989 ± 195.7 in the two-year follow-up. We compared the preoperative and postoperative ECD results and found a statistically significant difference (*P* ≤ 0.05). We also found a statistically significant decrease (*P* = 0.044) in the endothelium cell count between the postoperative follow-ups in the sixth and twelfth months. We did not find statistically significant differences among ECD results when compared with results of all postoperative follow-ups. Our results show that the most stressful times for the endothelium are the surgery itself and the early postoperative period. During further follow-ups, we observed endothelial cell density stabilization and only a small decrease in ECD, which was not statistically significant.

### 3.2. Uncorrected Distance Visual Acuity (UCDVA)

**Figure 4:**
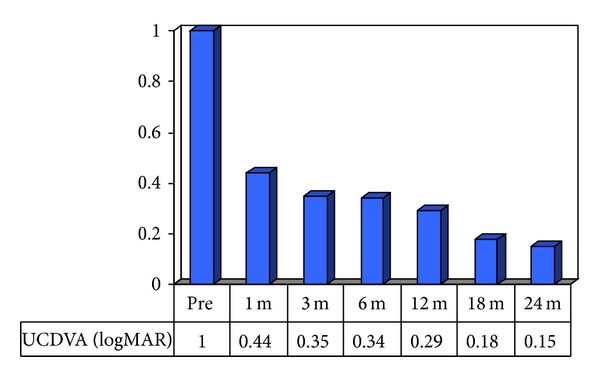
UCDVA results.

Another value that we evaluated over the follow-up period was uncorrected visual acuity (UCDVA). In our group of patients, the mean UCDVA was 1.0 ± 0.02 logMAR postoperatively, 0.44 ± 0.04 logMAR at one month postoperatively, 0.35 ± 0.04 logMAR at three months postoperatively, 0.34 ± 0.03 logMAR at six months postoperatively, 0.29 ± 0.04 logMAR at twelve months postoperatively, 0.18 ± 0.08 logMAR at eighteen months postoperatively, and 0.15 ± 0.10 logMAR at twenty-four months postoperatively. We observed a statistically significant improvement in UCDVA when compared with preoperative UCDVA and all further follow-up data. We also observed a statistically significant improvement in visual acuity when comparing the uncorrected at the one-month follow-up with that of the 18- and 24-month follow-ups (Figure 4).

### 3.3. Best Corrected Distance Visual Acuity (BCDVA)

**Figure 5:**
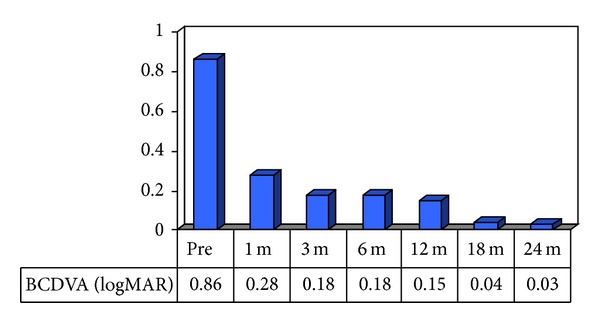
BCDVA results.

The best corrected visual acuity was 0.86 ± 0.02 logMAR postoperatively, 0.28 ± 0.04 logMAR at 1 month postoperatively, 0.18 ± 0.04 logMAR at 3 months postoperatively,0.18 ± 0.04 logMAR at 6 months postoperatively, 0.15 ± 0.04 logMAR at 12 months postoperatively, 0.04 ± 0.02 logMAR at 18 months postoperatively, and 0.03 ± 0.01 logMAR at 24 months postoperatively. We observed a statistically significant improvement in BCDVA when comparing preoperative BCDVA with all further follow-up data. We also observed a statistically significant improvement when comparing BCDVA the first month after surgery and at follow-up checks 18 and 24 months after surgery (Figure 5).

## 4. Discussion

PLK is becoming a gold-standard technique in the treatment of corneal endothelial diseases, including endothelial dystrophies, pseudophakic bullous keratopathy and endothelial graft failures. Various grafting approaches to PLK have been introduced in the past few years. Three main types of posterior corneal lamellae (PCL) are currently used in PLK according to their histological structure-DSEK/DSAEK, DMEK and hybrid technique DMEK-S. 

The main disadvantages of these operations are either their technical demands (DMEK) or the decrease in postoperative visual function due to the presence of interlamellar opacities (DSEK/DSAEK) [12, 15]. For this reason, the hybrid technique DMEK-S seems to be an ideal compromise, eliminating both disadvantages of the techniques mentioned previously. In comparison with that of other published data, endothelial cell loss immediately after surgery is higher [9, 11, 12, 16]. This is probably associated with a higher rebubbling rate. The high rebubbling rate is the second most serious disadvantage of this method. Theoretically, the viscomaterial used during the preparation of the lamella could be a cause of the higher rate of lamella detachment. Despite this, we suppose that its influence is very low, because we used only a thin layer of the viscomaterial exclusively in the central bending of the lamella. The viscomaterial causes a higher rebubbling rate only when present in the interface. Our explanation of the higher rebubbling rate is that the transition between the central bare Descemet membrane and the stromal rim in the periphery of the lamella is not completely smooth. There is always a small amount of fluid that can contribute to an increased incidence of lamella detachment.

In order to adopt this new technique in other practices, it is necessary to improve the high failure and rebubbling rate. Consequently, it may become the method of choice for many surgeons because of the ease of handling the lamella and the excellent visual results. The preparation of the lamella on the premises of the eye tissue bank, avoiding the risk of destroying the cornea in the operating room, contributes to this being a method of choice.

## 5. Conclusion

In summary, DMEK-S combines the advantages of DSEK/DSAEK and DMEK. The central zone of bare Descemet membrane and endothelium allows for very good visual outcomes, and the peripheral rim allows for better manipulation of the lamella during implantation. It is an effective method of treating the endothelial dysfunction of various etiologies, but the high complication rate needs to be addressed before widespread implementation of the technique in the future.

## Supplementary Material

This video shows combined DMEK-S and cataract surgery. The preparation of the donor cornea is shown at the beginning, followed by the main- and side- port incisions and the injection of the viscoelastic solution. The continual curvilinear capsulorhexis (CCC) is performed before descemethorhexis. Facoemulsification and lens implantation are done without the use of any viscoelastic material. The lamella is implanted into the anterior chamber using the Solution Implantation Technique and is well centered. After centration, the lamella is fixated to the recipient cornea using an air bubble.

## Figures and Tables

**Table 1:** Indications for DMEK-S surgery in our group.

Preoperative diagnoses	Number
Bullous keratopathy	27
Fuchs endothelial dystrophy	15
Posterior polymorphous corneal dystrophy	7
Endothelial failure after previous PLK	22

Total	71
